# Physical Presence during Moral Action in Immersive Virtual Reality

**DOI:** 10.3390/ijerph18158039

**Published:** 2021-07-29

**Authors:** Sylvia Terbeck, Jaysan Charlesford, Heather Clemans, Emily Pope, Aimee Lee, Joshua Turner, Michaela Gummerum, Bettina Bussmann

**Affiliations:** 1 School of Psychology, Liverpool John Moores University, Byron Street, Liverpool L3 3AF, UK; 2 School of Psychology, Plymouth University, Drake Circus, Plymouth PL4 4AG, UK; jaysan.charlesford@plymouth.ac.uk (J.C.); h.clemans@live.com (H.C.); emily.pope1994@live.co.uk (E.P.); aimeelee.1992@yahoo.com (A.L.); i7461944@bournemouth.ac.uk (J.T.); 3 Department of Psychology, University of Warwick, Gibbet Hill, Coventry CV4 7AL, UK; michaela.gummerum@warwick.ac.uk; 4 Department of Philosophy, Salzburg University, Franziskanergasse 1, 5020 Salzburg, Austria; bettina.bussmann@sbg.ac.at

**Keywords:** virtual reality, moral judgments, moral reasoning

## Abstract

Research on morality has focused on differences in moral judgment and action. In this study, we investigated self-reported moral reasoning after a hypothetical moral dilemma was presented on paper, and moral reasoning after that very same dilemma was experienced in immersive virtual reality (IVR). We asked open-ended questions and used content analysis to determine moral reasoning in a sample of 107 participants. We found that participants referred significantly more often to abstract principles and consequences for themselves (i.e., it is against the law) after the paper-based moral dilemma compared to the IVR dilemma. In IVR participants significantly more often referred to the consequences for the people involved in the dilemma (i.e., not wanting to hurt that particular person). This supports the separate process theory, suggesting that decision and action might be different moral concepts with different foci regarding moral reasoning. Using simulated moral scenarios thus seems essential as it illustrates possible mechanisms of empathy and altruism being more relevant for moral actions especially given the physical presence of virtual humans in IVR.

## 1. Introduction

Casebeer and Churchland [1] defined moral reasoning as “… a series of acts that result in a conclusion about what one ought to do or think” (p. 16). To study and illustrate human moral decision-making, most researchers use theoretical moral dilemmas, such as the trolley dilemma, in which one needs to decide whether to divert a trolley on an alternative track which would kill one man, but save five other people on the original track. A second “up close and personal” version of this dilemma involves pushing (and thereby killing) a person in front of the trolley as a means to prevent five people from being killed further down the track [2]. Typically, people choose to switch tracks in the “impersonal” trolley version to save the five; this has been termed as a utilitarian decision. In contrast, most people refuse to kill the one person by pushing him in front of the trolley; a deontological decision [3]. However, these moral decisions only have a binary outcome: Is it morally acceptable; ‘yes’ or ‘no’? Thus, the original trolley dilemma gives information about the moral decision, but not about the moral reasoning process that participants might engage in when they make this decision.

There has been a long tradition of investigating moral reasoning. Dating back to 1958, Kohlberg [4] assessed the moral development of children based on a model of moral reasoning. According to this theory, children’s moral reasoning progresses through several stages, such as developing from being authority depended (i.e., something is morally wrong because a person of authority has decided this) to more independent moral thought (i.e., something is morally wrong as it intrinsically neglects values in society agreed upon as a social code). However, this theory is mostly related to child development and has also been criticized on several grounds, such as choosing only male participants in the original longitudinal study of moral reasoning and neglecting the influence of emotional reactions, which might additionally guide moral decision making [5].

Indeed, the idea that ‘inborn’ emotional or intuitive responses might play a role in moral judgments was discussed by Turiel [6], who found that three-year-old children could already distinguish between conventional (i.e., something goes again a social convention) and moral transgressions. According to the philosophy of sentimentalism, emotions are suggested to play a causal role in moral judgments [7]. Previous research has also determined that there might be associations between emotional arousal and moral decisions. For example, Scher [8] found that anger and sadness were related to judgments of injustice and immorality. Blair [9] conducted a large number of studies and determined that emotional arousal and empathy might play a central role in moral judgments. Indeed, it has been postulated that people’s main moral intuition (i.e., ‘‘strong immediate moral belief’’—Sinnott-Armstrong [10], p. 47) in moral dilemmas is to avoid harming others at all costs (i.e., interpersonal harm aversion). Interpersonal harm aversion is thought to be rooted in empathic concern for others’ welfare [11,12,13]. Empathic concern, developed early in ontogeny, has been considered a necessary foundation of human morality [12,13].

However, most of these previous studies have focused on using theoretical moral vignettes. Indeed, for example the original paper-based trolley dilemmas measure people’s theoretical moral decisions; they do not reflect a behavioural decision (an actual moral action); “would someone … actually resort to this course of action when the full repertoire of contextual features comes into play?” [14] (p. 95). We recently developed a novel, state-of-the-art, immersive virtual reality paradigm to study moral behaviour in a most realistic animation [15]. We found that when asked if they would perform the action on paper and pencil (moral decision), only 10% of participants endorsed a utilitarian response (i.e., most participants said they would not push the man). However, in the virtual reality footbridge dilemma 70% actually performed the action (moral action). Further studies have supported and extended this research [16], showing for example that psychopath show intact moral decision but impaired moral action [17]. While these studies indicate that moral decision and moral actions have different outcomes, it is not clear whether they are based on different processes. In particular, do different moral reasoning processes underlie moral judgment (on paper and pencil) and moral action (in virtual reality)?

This study investigated self-reported moral reasoning in both; moral decision and moral action. Furthermore, interpersonal harm aversion, and empathic concern might be particularly highly elicited and mentioned in moral reasoning when being presented with a moral dilemma in IVR. Recent developments in IVR technology allow re-creating real-life contexts to measure actual behavioural responses in a controlled but realistic, ecologically valid setting [18]. Thus, IVR creates an illusion of reality, a feeling of presence and “being there” [18]. Indeed, much research has shown that participants respond to IVR as they would respond in real life. For example, people get aggressive when provoked by virtual humans [19], people get emotionally engaged in emergency scenarios [20], and can indeed feel empathy and exhibit pro-social behaviour towards virtual humans [21]. IVR was even promoted as a tool to teach individuals empathy, as it has been shown that IVR interventions were able to increase individual’s level of empathy and reduce anti-social behaviour [22]. In this study, we thus hypothesised that moral reasoning after moral action in IVR would show more person centered and empathic/altruistic reasons compared to theoretical moral decision making on a paper pencil measure. Specifically, we suggest that moral reasoning processing differences in IVR and paper/pencil would occur independent of the moral decision that is being made.

## 2. Materials and Methods

### 2.1. Participants

A total of 107 participants, 71 female, Mage = 24.29, SD = 10.37, participated in this study; we stopped recruitment after reaching 60 participants in the IVR, 47 participants in the paper-based condition. The current lab-based interview study tried to achieve a large sample size of participants, who were all face-to-face interviewed, making it a large sample for a content analysis research. We used a quasi-experimental design in which participants signed up for a virtual reality session or a questionnaire-based study.

### 2.2. Materials and Procedure

After informed consent, participants in the paper-based condition read the moral dilemma vignette of the footbridge dilemma and were then asked: “is it morally acceptable to perform the action?”. Answer options were “yes” or “no”. In the IVR condition, participants experienced the footbridge dilemma in the immersive environment. The design closely followed Francis et al. [15]. Participants were wearing the Oculus Rift 3D Immersive Virtual Reality Headset, and they responded to the dilemma with a joystick, requiring the action to push the man or not. In both conditions, participants then underwent an open-ended interview and their responses to the open-ended questions were written down verbatim (answers mostly included 1–2 sentences). The following open-ended questions were asked:

(a) In terms of morality and moral choices, do you believe you made the right decision? (Part 1) And why? (Part 2)
(b) How difficult was it for you to make that decision?
(c) Was your decision largely emotional or intellectual? (Part 1) Can you expand just a little on that? (Part 2)
(d) At the point that you were prompted to make a decision, what emotion or emotions did you feel, if any?
(e) Can you identify factors in your background which influenced your decision?
(f) Given the same situation, this time in real life, would you make the same decision? (Part 1) And why? (Part 2)

Two independent researchers inspected the answers and developed categories following the standards of content analysis [23]. The emerging categories were mutually exclusive, homogeneous, and exhaustive. For example, the categories for question (a) included the following (for the categories for all other questions, please see Supplementary Material);

(a) Greater good (logical reasoning);
(b) Accountability/responsibility of the participants themselves (thinking about personal consequences);
(c) Accountability/responsibility of the people on the line/or man on the bridge (thinking about consequences/intentions of people involved);
(d) Unsure (error/impulse);
(e) Realism/realistic (thinking about the situation as if it was real);
(f) Connection/personalization: personal relationship to the people involved (if they knew the workmen/large man);
(g) Fatalism;
(h) Believe there is no right or wrong answer/not their decision to make;
(i) Alternative solutions.

Table 1 gives an overview of each category and example statements.

The developed categories emerged from the participants responses and were thus formed following the answers participants gave towards the question.

Note, that we only used one participants’ first comment for the analysis. Specifically, in some cases, a participant might have made a longer reply, mentioning several reasons which could be assigned to multiple categories; in which cases we analyzed the first thing they said. This was due to the fact that participants varied in the number of reasons they gave, thus leading to missing data if the second or third response were also included. Subsequently, inter-rater reliability was determined between two independent raters using Cohen’s Kappa. The mean inter-rater reliability for participants’ assessment of whether their response was ‘right’ was excellent, K = 0.87, 95% CI [0.76, 0.95], *p* < 0.001, and categorisation of moral reasoning was substantial, K = 0.72, 95% CI [0.62, 0.82], *p* < 0.001.

## 3. Results

### 3.1. Most Common Reasons in Moral Judgment and Moral Action

**Figure 1:**
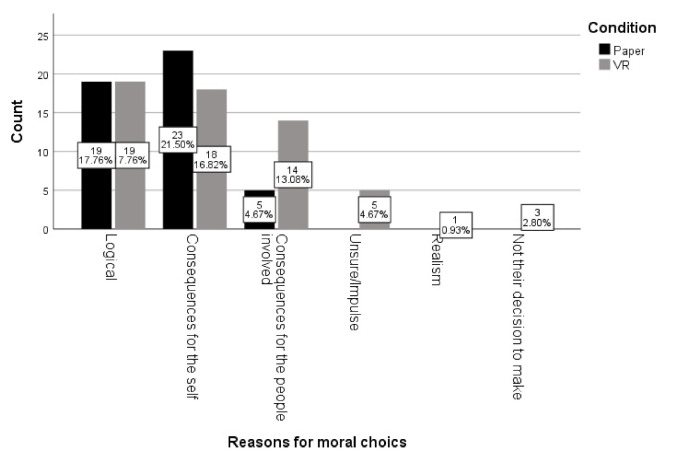
Differences in VR and paper version in moral reasoning (why did you make this choice?).

In both, the paper (76.6%) and VR (70.0%) version, most people stated that they thought they made the right decision (i.e., when they decided if it was morally acceptable or not to sacrifice one person in order to save five) (X2(2) = 0.58, *p* = 0.52). However, there was a significant difference in the answers to the questions why they made that choice between the paper and the VR version X2(5) = 12.48, *p* = 0.029) (See Figure 1).

As shown in Figure 1, participants in the VR version gave “thinking about the consequences of the people involved” as an answer more than in the paper version. They also named “error/impulse” as a reason, whilst no one mentioned this in the paper version.

**Figure 2:**
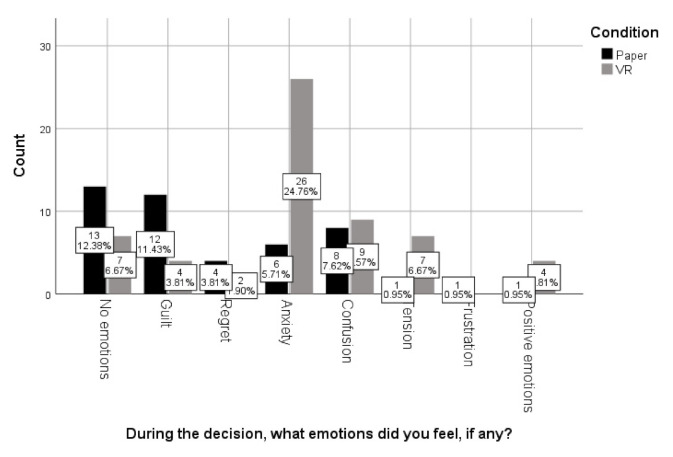
Differences in VR and paper version in moral reasoning (why did you make this choice?).

Regarding the question how difficult it was to make this choice, most people (36.2% paper, 26.7% VR) said that it was fairly difficult, with no significant differences between the conditions (X2(7) = 8.27, *p* = 0.31). In the paper version, 38.3% of participants said that their decision was intellectual, 40.4% said it was emotional, and 21.3% said it was a bit of both. Further, there was no significant difference to what people answered after the VR version on whether their decision was emotional, intellectual or a bit of both (41.7%; 21.7%; 36.7%) (X2(2) = 5.26, *p* = 0.07). However, when subsequently asked the next question to expand on this further, similarly to question one, participants in the VR condition showed a significantly different answer pattern (X2(7) = 14.34, *p* = 0.045), again mentioning thinking about the consequences of the people involves, their relatedness to the people, error/impulse more than on the paper version. Furthermore, there was a significant difference in the self-reported emotions for the paper and the VR version (X2(7) = 25.10, *p* = 0.001) (See Figure 2).

As shown in Figure 2, participants reported to feel greater anxiety, tension and confusion in the IVR condition than in the paper version, where they reported stronger feelings of sadness and guilt, or no emotions. When asked which factors had influenced their decision most people reported their upbringing (40.4% in the paper version), with no differences between conditions (X2(6) = 10.45, *p* = 0.11), 50% of participants in IVR stated that they would make the same decision if this was real life. A total of 57% said this in the paper version, and there were no significant differences between conditions (X2(2) = 4.07, *p* = 0.13). However, when asked to elaborate on this further, we found a near significant difference in the category pattern for IVR and paper (X2(9) = 15.25, *p* = 0.084). For instance, 78.3% of participants in the paper condition referred to consequences for themselves (compared to 61.7% in VR). However, 13.8% of participants in the IVR condition referred to consequences of the people involved in the dilemma, but no-one in the paper condition mentioned this. Finally, most participants in the paper and IVR condition (63% versus 53%) reported that this would be how they would respond to moral dilemmas in general.

### 3.2. Differences in Reasoning for Deontological Versus Utilitarian Actions

In the paper version 70.2% regarded the action to push the stranger as morally unacceptable. In the VR version more people (43.3%) actually performed the action. However, this difference was not significant (X2(1) = 2.07, *p* = 0.17). In the paper version, there was no significant difference in whether participants thought they made the right choice, depending on whether they made a utilitarian of deontological decision (X2(2) = 1.47, *p* = 0.48). However, there was in VR (X2(2) = 8.71, *p* = 0.013). In VR, 26.90% thought they made a wrong choice if they made a utilitarian choice, but only 2.9% thought they made a wrong choice with their inaction. Furthermore, most people (81.6%) gave utilitarian reasons for utilitarian decisions in both conditions (i.e., Code 1 = greater good/logical reasoning for the question of why they made this choice (Question 1B) (X2(1) = 49.17, *p* < 0.001)).

### 3.3. Predicting Conditions from Moral Reasoning

Using logistic regression, we determined if the moral reasons could predict the condition (0 = text based moral judgment versus 1 = VR based moral action). We used Question 2 “how difficult was it for you to make that decision?” (1 = very easy; 8 = very difficult), as well as four dummy coded variables which correspond to questions Q1a, Q1b, Q3a, Q4) (see Supplementary Material, for the original and the dummy coded scales).

We found the regression model to be near significant (X2(5) = 11.05, *p* = 0.05), with 38% of the variance being explained (Nagelkerke R Square). Specifically, the question “why did you make this moral choice?” was a near significant predictor (*p* = 0.05) with an odds ratio of 7.02 and an estimated b-value of 1.5. This means that referring to the consequences for other people (rather than the self) makes it seven times more likely that the participant had experienced the moral choice in VR (see Table 2).

## 4. Discussion

In this study, we investigated the moral reasons people used for justifying their moral decisions in a paper-based moral dilemma and their moral action in an IVR moral dilemma and found that reasons were different; in the paper-based condition, participants justified their decisions with moral rules affecting themselves, whilst participants in IVR referred to the people actually involved in the dilemma. Tassy et al. [24] suggested that the difference might be due to the fact that both outputs are part of separate processes (separate process hypothesis), and that judgments and behaviours are made from different perspectives, and our findings support this. Patil et al. [14] proposed that the saliency in action condition may have been generated by the ability to see potential victims. Indeed, immersive virtual reality (IVR) can be considered conceptually as a system that transforms their actions with reference to an exactly determined alternative reality. In our study, we also found that greater anxiety, stress and impulse were reported in IVR. Differences between self-reported action on paper-pencil and actual moral behaviour goes back historically to early studies by Milgram, who significantly found that participants reported on paper that they would never administer electric shocks to another person in order to follow an authority figure. However, in the actual experiment many participants did indeed show obedience [25,26]. We speculated that such differences in theoretical moral judgment and actual moral behaviour might be cause by a multitude of reasons, including possible impression formation and social desirability biases when self-reporting moral judgments [27].

In a recent study, Farsides, Sparks and Jessop [28] investigated the self-reported reasons behind theoretical moral judgments. Participants in an online survey responded towards six hypothetical moral dilemmas and were then presented with a questionnaire which included several reasons for action/inaction which the authors had pre-developed. The authors found that for the footbridge dilemma the most common motives were (in descending order): participants’ feelings, the nature of the action, identity, wanting to do the right thing, consequences for the self, relationships, and utilitarian concerns. Our study confirms these findings, detecting similar categories with an open interview approach. However, our study expands the previous one, as we have been investigating moral reasoning not only after theoretical moral judgments, but compared it to reasoning after active moral action in VR.

Previous research has determined that using virtual reality allows to create scenarios, which are more closely related to reality than merely reading about a situation and imagining it [19]. Indeed, it was determined that participants in VR experienced ‘presence’, defined as a feeling as if they were really there in that situation [19]. In addition, in our pervious study, participants also showed increased heart rate when they are placed in the trolley moral dilemmas situation in VR, compared to reading about it on paper, suggesting possibly greater emotional involvement in the situation [15]. Furthermore, in this previous study, participants reported that they regretted their moral choice in VR afterwards, which they did not report on the vignette version, also indicating possibly greater emotional involvement [15]. Previous studies in social psychology, that have to date been deemed unethical to repeat, such as the Milgram experiment, have been successfully re-created in VR [26]. In the VR study, it was found that similar to the original study in reality, participants delivered high electric shocks to virtual humans when ordered to do so, even when they reported this to be morally unacceptable in theory on a self-report questionnaire [26]. This indeed, also suggests that VR might deliver a new avenue to study moral realistic versions of moral behaviour. Rather than using the artificial version of the footbridge dilemma, a scenario which is unlikely to occur in real life, future studies might investigate moral situations in VR which are more realistic, possibly also involving active benevolence to others rather than harm avoidance. However, an IVR version of a theoretical paper-and-pencil task is off course different in many regards, for instance the aspect of seeing an interacting with the avatars might strongly contribute to the compassion and empathy elements. Thus, it remains unclear if the effect is attributable to the immersive aspect of the IVR, and future studies might incorporate a non-immersive desktop or 2D version of the task to investigate this further. Additionally, the response modes of IVR and paper-pencil version were different; specifically, on paper participants were asked if it was morally acceptable whereas in IVR participants actually performed the action. Future studies might investigate moral reasoning differences if participants are asked about their decision after merely encountering the IVR dilemma (i.e., without actually having to perform it).

A further limitation might be that participants are not willing or able to accurately report their reasons for action in the subsequent interview, however there is no reason to believe that participants would be more or less willing to report reasons if they had been doing the paper or the VR version. Furthermore, we used a quasi-experimental design, which could limit its conclusions. Additionally, future studies might include individual differences measures, such as empathic concern, in order to determine if personality differences might mediate the effects. Finally, as a practical application, IVR tasks might be used for moral education, making individuals aware of their moral responses, such as in school, universities, or in health professions.

## 5. Conclusions

Overall, our study suggests that moral action and moral decision are not just associated with different outcomes, but that moral judgment and moral action might also involve separate reasoning processes. Furthermore, moral behaviour might be more guided by emotional processes, such as empathic concern. Empathic concern and altruism might be particularly high when being immersed into the situation in virtual reality, allowing for “presence” of virtual humans. Thus, future research could investigate altruism more when employing IVR environments.

## Figures and Tables

**Table 1:** Illustrative table for each statement in the content analysis.

Category	Illustrative Statement
Greater good (logical reasoning)	Yes, because it is better to save more people compared to just one person.
Accountability/responsibility of the participants themselves	Yes. I think if I got involved and pushed, I would be committing murder
Accountability/responsibility of the people on the line/or man on the bridge	Yes, people on the track were already there and it would not have been fair to put the fat man in danger.
Unsure (error/impulse)	Do not know. Why are the people on the track?
Realism/realistic	It is the laws of physics that the trolley was going to the five men.
Connection/personalization: personal relationship to the people involved	The five put themselves at risk. I am not attached to any.
Fatalism	By doing nothing, whatever happened, happens.
Believe there is no right or wrong answer Alternative solutions	Hard to choose, I do not know what would be justified: saving one or saving more. Is there a slight chance the group may have jumped to safety at last minute?

**Table 2:** Predictors in the logistic regression model with independent variable of condition (moral decision versus moral action).

Predictors	B	Wald	Significance	Odds-Ratio
Did you make the right choice?	1.45	0.80	0.37	4.27
Concern about the self-versus—other	1.95	3.72	0.54 *	7.02
Emotion versus Intellect	−1.53	2.07	0.15	0.22
How difficult was the choice	−0.15	0.56	0.46	0.86
No emotion versus emotion report	−2.31	2.27	0.13	0.10
Constant	−2.68	0.58	0.45	0.70

* is significant at *p* = 0.05.

## Data Availability

Data supporting reported results can be found at available online: https://osf.io/qwxd9/?view_only=28299bf5f1844165aebf82842051b2e5 (accessed on 8 July 2021).

## Supplementary Materials

The following are available online at https://www.mdpi.com/article/10.3390/ijerph18158039/s1, codes for the content analysis including dummy codes.
