# Supplementary material for: Physical Presence during Moral Action in Immersive Virtual Reality
Source: Int J Environ Res Public Health. 2021 Jul 29;18(15):8039. doi: 10.3390/ijerph18158039 (PMC8345728; doi:10.3390/ijerph18158039)
Supplement: Supplementary file 1 [file ijerph-18-08039-s001.zip › ijerph-1259644-supplementary.pdf]

International Journal of
Environmental Research and Public Health

MDPI

Supplementary Material: Codes used in the content analysis of the data

Question 1

Section A: Did you think you made the right choice

1) Yes
2) No
3) Unsure (Don't know, Error/Impulse)

Dummy code:

1) Yes
2) No

Section B: WHY

1) Greater Good (*Logical reasoning)*
2) Accountability/Responsibility of the participants themselves (*thinking about personal consequences)*
3) Accountability/Responsibility of the people on the line/or man on the bridge (*thinking about consequences/intentions of people involved)*
4) Unsure (Error/Impulse)
5) Realism/Realistic (*Thinking about the situation as if it was real)*
6) Connection/Personalization: Personal relationship to the people involved (*If they knew the* workmen/fatman)
7) Fatalism
8) Believe there is no right or wrong answer/not their decision to make
9) Alternative solutions

Dummy code:

1) Accountability/Responsibility of the participants themselves (*thinking about personal consequences)*
2) Accountability/Responsibility of the people on the line/or man on the bridge (*thinking about consequences/intentions of people involved)*

Question 2 - How difficult was it for you to make that decision?

1) Very easy
2) Easy
3) Fairly easy
4) Medium / 50/50
5) Neither
6) Fairly difficult
7) Difficult
8) Very difficult

Question 3

Section A: Was your decision largely emotional or intellectual?

1) Intellectual
2) Emotional
3) Mixture of both

Dummy code:

1) Intellectual
2) Emotional

Section B: Can you expand just a little on that?

1) Greater Good (*Logical reasoning)*
2) Accountability/Responsibility of the participants themselves (*thinking about personal consequences)*
3) Accountability/Responsibility of the people on the line/or man on the bridge (*thinking about consequences/intentions of people involved)*
4) Unsure (Error/Impulse)
5) Realism/Realistic (*Thinking about the situation as if it was real, physical actions etc.)*
6) Connection/Personalization: Personal relationship to the people involved *(If they knew the workmen/fatman / giving them characterisation)*
7) Fatalism
8) Believe there is no right or wrong answer/not their decision to make
9) Alternative solutions
10) Empathy

Question 4 - At the point that you were prompted to make a decision, what emotion or emotions did you feel, if any?

1) No emotions
2) Guilt
3) Regret *(sadness)*
4) Anxious *(worry, stress, pressure, fear, panic)*
5) Confusion *(Uncertainty, hesitation, torn, indecisive, reluctant)*
6) Under pressure (*Tension)*
7) Frustration
8) Positive emotions (*Smug, excited, calm, natural)*

Dummy code:

1) No emotions
2) Emotions (any 2-8)

Question 5: Can you identify factors in your background which influenced your decision?

1) Media (*News, video games)*
2) Beliefs (*Religion, politics and culture)*
3) Personality (*Hobbies, traits)*
4) Upbringing *(Family background)*
5) Education and work experience
6) Life experiences (*Seeing death before)*
7) None identified

Question 6

Section A: Given the same situation, this time in real life, would you make the same decision?
1) Yes
2) No
3) Unsure (Don't know, Error/Impulse)

Section B: WHY?
1) Greater Good (*Logical reasoning)*
2) Accountability/Responsibility of the participants themselves (*thinking about personal consequences)*
3) Accountability/Responsibility of the people on the line/or man on the bridge (*thinking about consequences/intentions of people involved)*
4) Unsure (don't know)
5) Connection/Personalization: Personal relationship to the people involved *(If they knew the workmen/fatman / giving them characterisation)*
6) Fatalism
7) Believe there is no right or wrong answer/not their decision to make
8) Alternative solutions *(self sacrifice, shouting out etc.)*
9) Difficult decision
10) Unlikely in real life (artificiality/fake/unrealistic situation)

Question 7

Section A: Putting to one side now the fact that this was a Virtual Reality experiment, would the decision you made be consistent with how you would respond to moral dilemmas in real life?
1) Consistent
2) Inconsistent
3) Not sure

Section B: Can you explain very briefly
1) Bystander *(Non-interventionism or inertia/inaction)*
2) Greater Good
3) Depends on situation
4) Emotional influences (*Fair/Unfair)*
5) Not their decision to make
6) Alternative solutions *(self sacrifice, shouting out etc.)*

Section C: perhaps with an example?
1) Aggression (*War, terrorism, bullying*)
2) Personal *(Pets, family, friends, relationships, views)*
3) Media *(TV, news)*
4) Rules *(Law etc)*
5) Work experience
6) Alternative moral dilemma examples

Question 8:
1) Yes
2) No
